# Bonding dissimilar polymer networks in various manufacturing processes

**DOI:** 10.1038/s41467-018-03269-x

**Published:** 2018-02-27

**Authors:** Qihan Liu, Guodong Nian, Canhui Yang, Shaoxing Qu, Zhigang Suo

**Affiliations:** 1 John A. Paulson School of Engineering and Applied Sciences, Kavli Institute for Bionano Science and Technology, Harvard University, Cambridge, MA 02138 USA; 2 State Key Laboratory of Fluid Power & Mechatronic System, Key Laboratory of Soft Machines and Smart Devices of Zhejiang Province, and Department of Engineering Mechanics, Zhejiang University, 310027 Hangzhou, China

## Abstract

Recently developed devices mimic neuromuscular and neurosensory systems by integrating hydrogels and hydrophobic elastomers. While different methods are developed to bond hydrogels with hydrophobic elastomers, it remains a challenge to coat and print various hydrogels and elastomers of arbitrary shapes, in arbitrary sequences, with strong adhesion. Here we report an approach to meet this challenge. We mix silane coupling agents into the precursors of the networks, and tune the kinetics such that, when the networks form, the coupling agents incorporate into the polymer chains, but do not condensate. After a manufacturing step, the coupling agents condensate, add crosslinks inside the networks, and form bonds between the networks. This approach enables independent bonding and manufacturing. We formulate oxygen-tolerant hydrogel resins for spinning, printing, and coating in the open air. We find that thin elastomer coatings enable hydrogels to sustain high temperatures without boiling.

## Abstract (web summary)

Hydrogels and hydrophobic elastomers of various shapes are difficult to bond in arbitrary sequences. Here the authors mix silane coupling agents into a precursor to form hydrogel resins with robust properties that can be spun, coated and printed in air.

## Introduction

An integrated circuit achieves its function by integrating dissimilar components, and so does a living organ. A family of recently demonstrated devices mimics the functions of neuromuscular and neurosensory systems—actuating, sensing, and signaling—by integrating hydrogels and elastomers. The hydrogels function as stretchable, transparent, ionic conductors. The elastomers function as stretchable, transparent dielectrics. The elastomers also function as seals to retard dehydration when the devices are in the open air, or to retard the exchange of solutes when the devices are in an aqueous environment. To function as dielectrics and seals, the elastomers must be hydrophobic, with low solubility and diffusivity of water. Demonstrated devices include transparent loudspeakers^1^, ionic skins^2–5^, ionic cables^6^, stretchable electroluminescent displays^7,8^, soft touchpads^9,10^, soft actuators^1,11,12^, and triboelectric generators^13^. In particular, a salt-containing and elastomer-coated hydrogel fiber mimics the myelinated axon as a fast conduit for electrical signals, and endures the wearing and washing conditions commonly associated with textiles^14^. Such an artificial axon may be used to develop soft touchpads and soft displays for wearable and washable smart clothes.

The emergence of these devices has posed a fundamental challenge: hydrogels and elastomers without covalent bonds have low adhesion energy (typically below 1 J/m^2^)^15^, far below the fracture energy of common hydrogels (typically around 100 J/m^2^), and tough hydrogels and elastomers (typically above 1000 J/m^2^)^16,17^. Existing bonding approaches demonstrate strong adhesion between hydrogels and elastomers, but are restricted to specific sequences of forming the networks. Gluing requires two preformed networks^4,18^. Grafting after surface activation requires forming one network on a preformed network^5^, which has only been demonstrated to graft a hydrogel on an elastomer, not graft an elastomer on a hydrogel. Copolymerization requires forming two networks together^19,20^, which has only been demonstrated for acrylate-based elastomer and hydrogel^19^. The restriction to the specific sequences of forming the networks fundamentally limits manufacturing capabilities.

Here we describe an approach to meet the challenge. We add silane coupling agents^21–24^ into the precursors of both hydrogels and elastomer. The silane coupling agents condensate after manufacturing and generate bonding across the interface. The idea is similar to placing a reactive inside different thermoplastics to improve adhesion^25–27^. Our approach improves adhesion independent of the sequence of forming the networks, and enables various manufacturing processes difficult or impossible to achieve using existing approaches. The bonding kinetics can be tuned by changing the temperature and pH, and by adding surfactants. This bonding approach enables capabilities not reported before. We formulate oxygen-tolerant hydrogel resins for spinning, printing, and coating in air. We find that elastomer-coated hydrogels sustain high temperatures without boiling. These capabilities open doors to new applications, such as soft touchpads and soft displays for smart clothes that one can wear, wash, and iron.

## Results

### Bonding mechanism

**Fig. 1:**
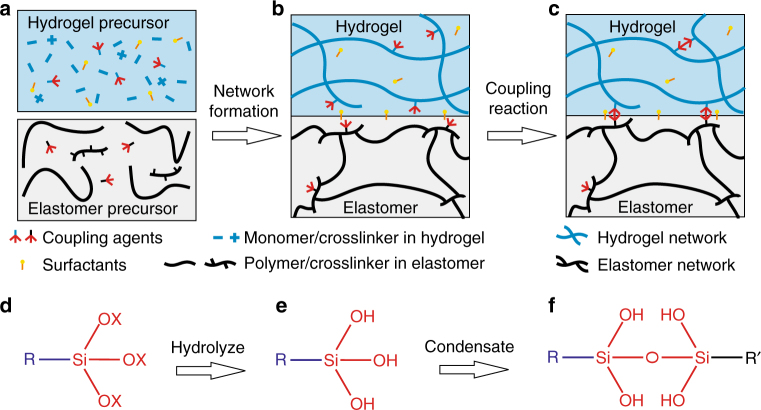
A hydrogel and an elastomer form covalent bonds after a manufacturing process. **a** Silane coupling agents are mixed into the precursors of a hydrogel and an elastomer separately. **b** During the formation of the two networks, the coupling agents are covalently incorporated into the networks, but do not condensate. **c** After a manufacturing process, the coupling agents condensate, add crosslinks in the individual networks, and form bonds between the networks. A surfactant may further promote adhesion. **d** Silane coupling agents hydrolyze and form **e** Silanol groups, which condensate to form **f** Siloxane bond

Silane coupling agents are added into the precursors of both the hydrogel and the elastomer (Fig. 1a). The coupling agents with a suitable functional group can be grafted or copolymerized into the networks (Supplementary Fig. 1). We tune the kinetics such that when the precursors form separate networks, the coupling agents copolymerize into the networks, but do not condensate (Fig. 1b). After a manufacturing step, the coupling agents condensate, add crosslinks inside the individual networks, and form bonds between the networks (Fig. 1c). In a silane coupling agent, a silicon atom links three hydrolyzable groups (e.g., hydroxy, acetoxy, chloro) and an organofunctional group (Fig. 1d). During the formation of a polymer network, the organofunctional group covalently incorporates the trialkoxysilane into the network. In the presence of water, the alkoxy groups hydrolyze into silanol groups (Fig. 1e). Subsequently, the silanol groups condensate to form a siloxane bond (Fig. 1f). Silane coupling agents with various choices of the organofunctional group are available, allowing our approach to be used in different polymer systems (Supplementary Table 1).

### Bonding different materials in different sequences

**Fig. 2:**
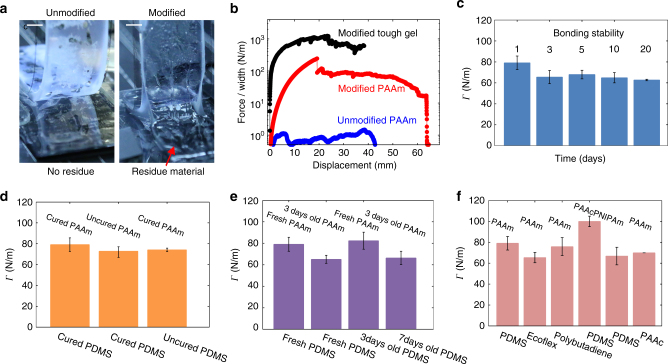
Bonding hydrogels and elastomers. **a** For unmodified PAAm and PDMS, the hydrogel peels off on the interface and leaves no residue on the elastomer. For silane-modified PAAm and PDMS, fracture occurs in the hydrogel, leaving residue on the elastomer. The scale bar is 5 mm. **b** The force–displacement curves for a silane-modified PDMS substrate bonded with various forms of PAAm: unmodified PAAm (blue), silane-modified PAAm (red), and silane-modified PAAm toughened by an interpenetrating polyacrylate network (black). **c** The adhesion energy drops slightly over the first few days as the coupling agents condensate, but stabilizes afterwards (*n* = 3–5). **d** Bonding is achieved for various sequences of forming the networks (*n* = 3–5). **e** Bonding is achieved even if the silane-modified samples are a few days old before contact (*n* = 5). **f** Bonding is achieved for various hydrogels and elastomers (*n* = 3–5)

We first bond a free-radical polymerized polyacrylamide (PAAm) hydrogel and an addition-cured polydimethylsiloxan (PDMS) elastomer. We mix different trialkoxysilanes into the precursors of the hydrogel and the elastomer, and form the two networks separately. The hydrogel precursor uses *α*-ketoglutaric acid as the photo-initiator, giving a pH ~3.5. We then place them in contact, seal the bilayer in a Petri dish, and keep it at room temperature for 1 day. Afterward, we measure the adhesion energy using the 90-degree peeling test (Fig. 2a, Supplementary Fig. 2). If the coupling agents are not added, or only added to one precursor, the adhesion energy is low (1.0 J/m^2^), and the hydrogel peels on the interface. If the coupling agents are added to both precursors, the adhesion energy is high (80.5 J/m^2^), and fracture occurs in the hydrogel.

**Fig. 3:**
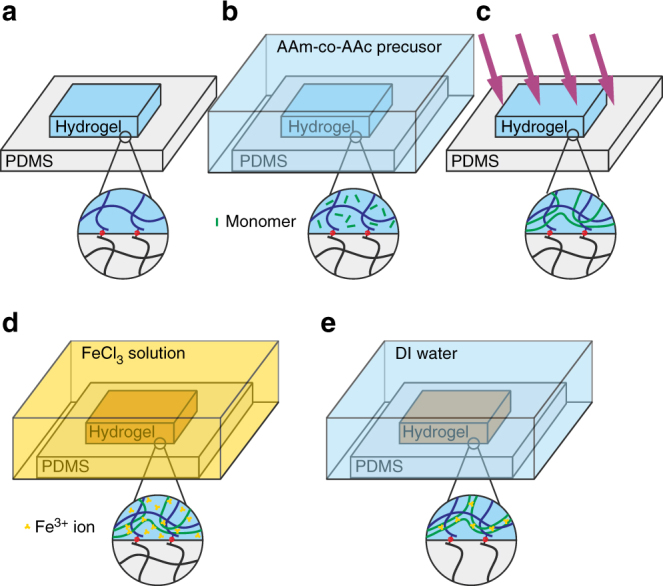
Toughening the silane modified PAAm-PDMS by infusing a dissipative interpenetrating network. **a** A silane-modified PDMS precursor is spin-coated on a glass slide. A silane-modified PAAm hydrogel is bonded on the silane-modified PDMS. **b** The bilayer is immersed in the precursor of the PAAm-co-PAAc hydrogel for 1 day. **c** The precursor polymerizes under UV lamp, but does not crosslink at this stage. **d** The sample is immersed in FeCl_3_ solution for 1 day. The Fe^3+^ ions form coordinate compounds with the carboxylic acid groups, which physically crosslinks the PAAm-co-PAAc network. The siloxane bonds between the PAAm and PDMS chains are strong bonds, and the coordination complexes are weak bonds. The hydrogel becomes brownish after the crosslinking. **e** The sample is then immersed in distilled water for 1 day to remove excess Fe^3+^ ions

Our bonding approach can readily demonstrate a fundamental principle in fracture mechanics. Achieving high fracture energy requires the synergy of strong and weak bonds. Fracture breaks only one layer of strong bonds, but breaks many more weak bonds, greatly amplifying the measured fracture energy. To demonstrate this principle, we form a siloxane-bonded PAAm–PDMS bilayer as before. We then infuse into the PAAm hydrogel a precursor that forms copolymers of acrylamide (AAm) and acrylic acid (AAc) (Fig. 3). The bilayer is immersed in an aqueous solution of FeCl_3_ to form coordination complexes of Fe^3+^ ions and carboxylic groups^28^. The siloxane bonds between the PAAm and PDMS chains are strong bonds, and the coordination complexes are the weak bonds. The peeling test of the toughened PAAm on PDMS gives an adhesion energy of 866.9 J/m^2^ (Fig. 2b). Still, fracture happens in the hydrogel instead of on the interface.

**Fig. 4:**
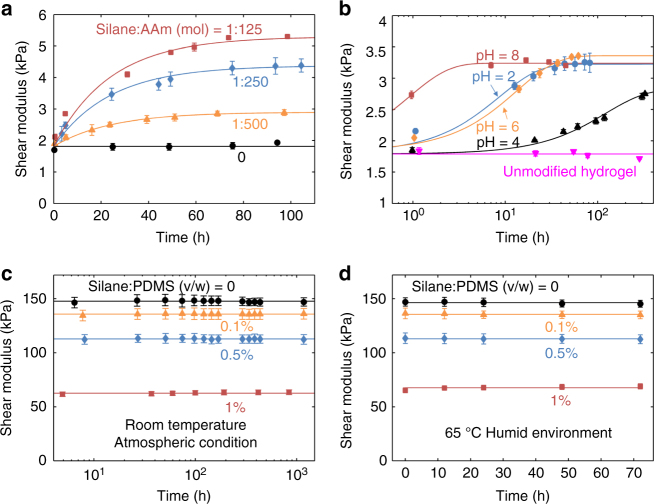
The kinetics of silane coupling agents in PAAm and PDMS. **a** The modulus of the PAAm hydrogel changes with time as the coupling agents condensate. The amount of coupling agent does not affect the initial modulus, but affects the modulus after condensation. **b** The rate of condensation of trimethyloxysilane inside a hydrogel depends on pH. **c** The modulus of the PDMS elastomer does not change with time, but decreases as the amount of coupling agent increases. **d** PDMS with different amounts of TEOVS is sealed in a Petri dish with drops of distilled water and kept at 65 °C in an oven. The modulus does not change over time (*n* = 3)

The stability of the bonding is confirmed by a series of peeling tests up to 3 weeks after bonding (Fig. 2c). The adhesion energy drops slightly over the first few days, but stabilizes as the condensation completes. The drop is explained by the increase of crosslinking density of the hydrogel as the coupling agents condensate (Fig. 4a), making the hydrogel stiffer and less tough.

Our approach bonds hydrogels and elastomers for various sequences of forming the networks (Fig. 2d, uncured hydrogel on uncured elastomer is demonstrated later with the development of a hydrogel resin). Our approach allows time for manufacturing before bonding. For example, we have bonded a 3-day-old hydrogel to a 7-day-old PDMS (Fig. 2e). The bilayers always fracture in the hydrogel, not on the interface. As noted above, the fracture energy deceases slightly because the intra-network condensation of the coupling agent increases the crosslinking density. The approach is generally applicable to various hydrogels and elastomers using commercially available silane coupling agents (Fig. 2f, Supplementary Fig. 1). Bonding between different hydrogels is also realized, e.g., between PAAm and polyacrylic acid (PAAc).

### Tuning stiffness and adhesion independently

The silane-modified networks enable us to study the kinetics of intra-network condensation. The initial PAAm network forms by the conventional crosslinker *N*,*N*-methylenebisacrylamide (MBAA). During the formation of the initial PAAm network, the coupling agents copolymerize into the polymer chains, but do not condensate, so that the shear modulus of the initial network is unaffected by the coupling agents (Fig. 4a). Subsequently, the coupling agents condensate and add crosslinks, so that the shear modulus of the PAAm hydrogel increases with time. The delay time is independent of the amount of the coupling agent. Mobile silanols in an aqueous solution condensate at a rate depending on the pH, and the lowest rate occurs around pH = 4^29^. We observe similar behavior for silanols fixed on the polymer chains in the PAAm hydrogel (Fig. 4b). In Fig. 2, *α*-ketoglutaric acid is used as the initiator to form PAAm hydrogels, resulting in a pH ~3.5 in the precursor.

In the PDMS elastomer, the coupling agents react with the conventional crosslinker polymethylhydrosiloxane (Supplementary Fig. 1b). The coupling agents compete with the conventional crosslinking process and lower the modulus of the network (Fig. 4c). Since PDMS is hydrophobic, the coupling agents rarely hydrolyze and condensate. We observe no change in the modulus of the PDMS at room temperature a month after curing, and no change for samples at 65 °C in a humid environment for 3 days either (Fig. 4d). For most practical purposes, the coupling agents in the PDMS can be treated as nonreactive.

In either the hydrogel or the elastomer, we can independently vary the amounts of the coupling agents and the conventional crosslinkers. These variables allow the independent tuning of the moduli of the networks and the adhesion between the networks.

### Promoting the adhesion with surfactant and temperature

The inter-network condensation of the coupling agents generates bonding. The coupling agents in the hydrogel side readily hydrolyze and condensate, but the coupling agents inside the PDMS do not. The different hydrolysis kinetics across the interface impedes bonding. In the limiting case, no bonding is possible if all the coupling agents in the hydrogel have condensated before one single coupling agent in the elastomer has hydrolyzed.

One way to improve the bonding is to add more coupling agents to the precursor of PDMS, but this wastes the coupling agents. Another way is to accelerate the hydrolysis and condensation of the trialkoxysilane in the elastomer by the tin-based catalyst^30–32^. This would be an efficient choice for most elastomers, e.g., polybutadiene (Fig. 2f). However, this catalyst would inhibit the crosslinking of the addition-cured PDMS^30^.

**Fig. 5:**
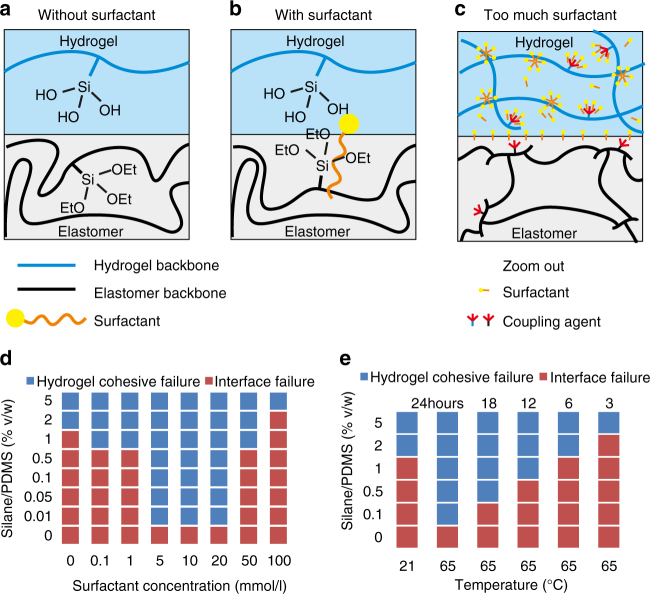
Effects of surfactant and temperature on adhesion. **a** The pre-hydrolysis trialkoxysilane on the elastomer chains is hydrophobic, and is hard to stick into the hydrogel. **b** The surfactant moves to the hydrogel–elastomer interface, and helps the trialkoxysilane on the elastomer chains hydrolyze and condensate. **c** When too much surfactant is added into the hydrogel, the surfactant–polymer complex covers the relatively hydrophobic coupling agent, which slows down the condensation reaction. **d** We use a matrix of peeling tests to study how the surfactant affects the amount of the coupling agent in the elastomer needed for strong adhesion. Bonding is considered successful if the peeling test causes a fracture inside the hydrogel, rather than on the hydrogel–elastomer interface. The default recipe uses 10 mmol/l SDS. **e** Higher temperature speeds up the bonding and reduces the amount of required coupling agent in the elastomer

We find two other ways to promote inter-network condensation. The first way is to add a surfactant to the precursor of the hydrogel. The pre-hydrolysis coupling agent on the elastomer chains is hydrophobic, and is difficult to stick into the hydrogel. The surfactant adsorbs at the hydrogel–elastomer interface, and helps the coupling agent on the elastomer chains get solvated and hydrolyzed (Fig. 5a, b). To test this hypothesis, we fix the amount of coupling agent in the hydrogel, and vary the amount of coupling agent added to the elastomer and the amount of surfactant added to the hydrogel. For each sample, a peeling test is performed after 1 day of contact. Bonding is considered successful if fracture occurs in the hydrogel instead of on the interface. Adding a suitable amount of surfactant sodium dodecyl sulfate (SDS) to the PAAm precursor reduces the required amount of coupling agent in the PDMS by two orders of magnitude (Fig. 5d). As we vary the amount of added surfactant, the coupling efficiency first increases then decreases. Above certain concentration, the surfactant aggregates with the polymer chain of the hydrogel^33^. Such aggregation hides the silanol groups in the hydrogel, and inhibits adhesion (Fig. 5c).

The second way is to increase the temperature during bonding. Additional thermal energy accelerates all the reactions. Consequently, within the same amount of time, more trialkoxysilanes on PDMS near the interface hydrolyze and condensate with the trialkoxysilanes on PAAm. At 65 °C, the required amount of coupling agent in PDMS reduces by one order of magnitude (Fig. 5e). Additionally, heating significantly reduces the time required for bonding.

### Oxygen-tolerant hydrogel resins

Free-radical polymerized hydrogels are widely used in bioengineering^34,35^, optics^36–39^, and soft ionic devices^1–13^. Since the free radicals can be quenched by oxygen, the synthesis of the hydrogel requires a sealed container or an inert atmosphere, which severely limits manufacturing capabilities. In addition, resins of certain ranges of viscosity are required in many manufacturing processes, such as printing, extrusion, rolling, and embossing. The viscosity of a monomer precursor solution is too low for these processes.

**Fig. 6:**
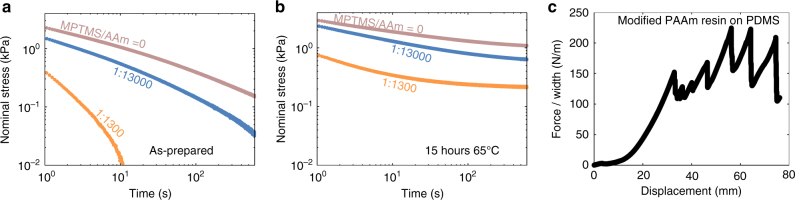
Oxygen-tolerant hydrogel resin that bonds with silane-modified elastomer resin. **a** Stress relaxation tests show that as the average chain length decreases by adding chain transfer agent, the stress relaxation becomes faster. **b** After 12 h at 65 °C, the polymers are crosslinked into a network by the condensation of silanol groups. Consequently, the sample can hold stress without relaxing. **c** Oxygen-tolerant resin allows uncured PAAm resin and uncured PDMS resin to be layered, cured together, and form robust bonding

We use trialkoxysilanes to formulate oxygen-tolerant hydrogel resins. We make silane-modified PAAm precursor as before, but remove the conventional crosslinker MBAA. The precursor is polymerized in a sealed container. The precursor becomes a viscous fluid right after curing, and its viscosity can be tuned (Fig. 6a) using a chain transfer agent, (3-Mercaptopropyl)trimethoxysilane (MPTMS) (Supplementary Fig. 3). Subsequently, the coupling agents condensate, crosslink the polymer into a network (Fig. 6b), and form bonding with neighboring silane-modified materials (Fig. 6c). The resin allows uncured hydrogel to bond with uncured elastomer, together with Fig. 2d we have demonstrated bonding hydrogel and elastomer in all possible sequences. The condensation is oxygen tolerant. Like a hydrogel, the kinetics of condensation in a resin can be tuned by pH and temperature, allowing the pot-life of resins from a few hours to a few days.

### Bonding in various manufacturing processes

As noted above, the kinetics of condensation (therefore bonding) can be tuned by changing the temperature and pH, as well as by adding surfactants and catalysts. Consequently, we can make the time scale for bonding to be much longer than the time scale for manufacturing (e.g., casting, assembling, printing, and coating). It is this separation in time scales that enables our approach to be generally applicable to various manufacturing processes. Since bonding happens after manufacturing, our approach is compatible with high-throughput manufacturing. For example, alternating layers of hydrogels and elastomers can be printed in a short time, and then removed from the printer to cure and bond. The post-printing cure does not occupy the printer and thus does not interfere with high-throughput printing. We next demonstrate our approach in various manufacturing processes that forms networks in different sequences.

**Fig. 7:**
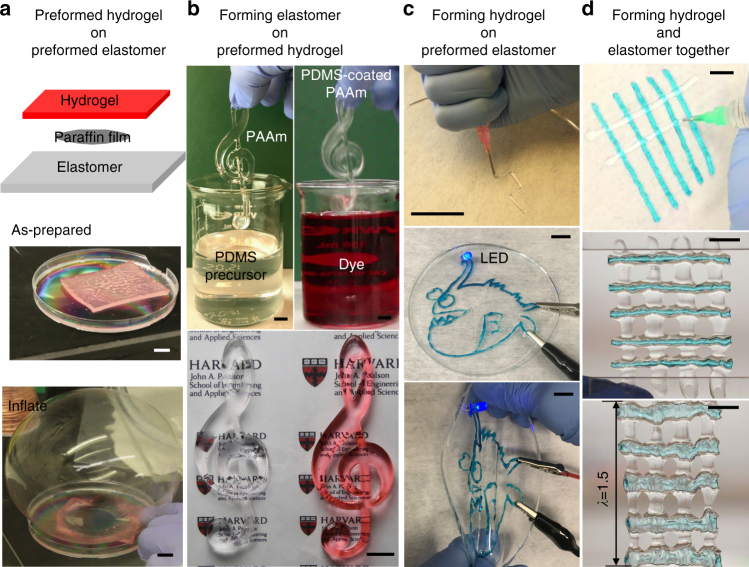
Manufacturing processes that involve various sequences of forming networks. **a** A preformed hydrogel network on a preformed elastomer network. A PAAm hydrogel and a PDMS elastomer are separately molded, and then placed in contact with a thin film of paraffin sandwiched in between. After curing, the contact region between the hydrogel and the elastomer forms bonds while the paraffin region does not. The bonding remains intact as a nozzle inflates the hydrogel into a balloon. **b** Forming an elastomer network on a preformed hydrogel network. A PAAm G-clef is molded, and then dip-coated with a thin layer of PDMS. The PDMS-coated PAAm is dyed in a bath. The color is readily washed away in clean water. By comparison, if a naked hydrogel is dyed in the bath, the color cannot be washed away. **c** Forming a hydrogel network on a preformed elastomer network. A PDMS film is cast first. On the PDMS film, a hydrogel ionic circuit with the shape of an anglerfish is written using a syringe. A light-emitting diode (LED) is switched on. The circuit remains adherent when the elastomer is stretched. **d** Forming a hydrogel network and an elastomer network together. A silane-modified PAAm hydrogel resin is colored with a blue dye and extruded on a Petri dish. Partially cured PDMS resin is then extruded into lines perpendicular to the hydrogel lines. After curing, the mesh withstands stretching. The scale bars represent 10 mm

First, the approach bonds preformed hydrogel and elastomer networks. The adhesion is strong enough to sustain different types of load, such as the peeling encountered in pneumatic actuator (Fig. 7a, Supplementary Movie 1). Among existing approaches to bond hydrogels and elastomers, gluing also bonds preformed polymer networks^4,18^. Our approach avoids interrupting the manufacturing to apply glue, does not introduce a glue layer between the interfaces.

Second, the approach works for an elastomer network formed on a preformed hydrogel network (Fig. 7b). We dip-coat a thin layer of elastomer over a G-clef-shaped hydrogel. The elastomer coating retards mass exchange between the hydrogel and the environment, and the bonding survives stretch and rub (Supplementary Movie 2). No previously existing approach can coat thin layers of elastomers on hydrogels of arbitrary shape.

Third, the approach works for a hydrogel network formed on a preformed elastomer network. A hydrogel is a stretchable ionic conductor. We can draw a soft ionic circuit on an elastomer (Fig. 7c). Among existing approaches, grafting after surface activation also works for this situation^5^. Our approach avoids interrupting the manufacturing for surface activation.

Fourth, the approach bonds two networks that are formed concurrently. Printing of hydrogel–elastomer composite requires dissimilar networks to be formed on top of each other in alternating sequence^3,40^. As a proof-of-concept demonstration, we syringe-print a PAAm–PDMS mesh (Fig. 7d). The mesh is stretchable after curing (Supplementary Movie 3). In principle, the crosslinking reaction of the polymer network can be much faster than the bonding reaction, and the rheological properties of the ink can be modified by long chain polymers or filler particles. Better printing resolution can be achieved by tuning the crosslinking kinetics and the rheological properties. Among existing approaches, copolymerization also prints multilayers of hydrogels and elastomers^19^. But copolymerization is only applied to acrylate-based hydrogels and elastomers. Our approach allows printing of various hydrogels and elastomers in arbitrary sequences.

**Fig. 8:**
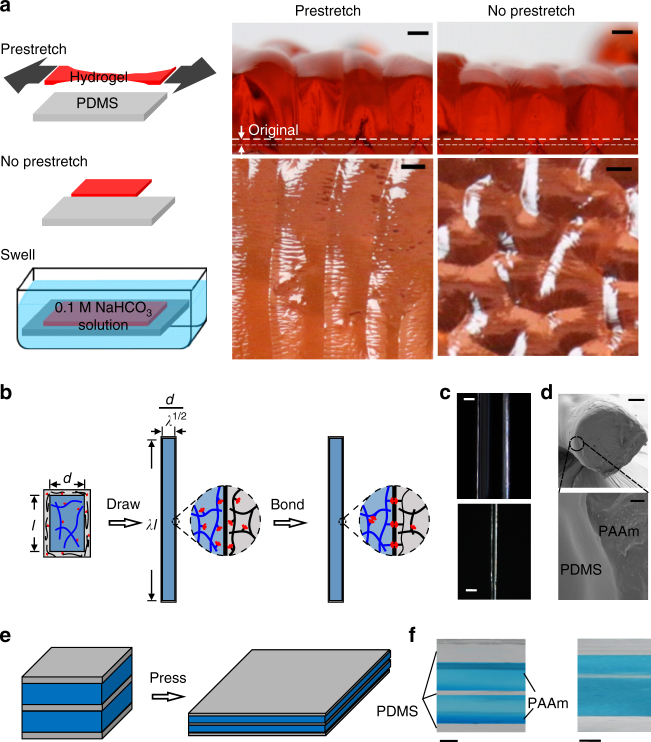
Mechanical manipulation during bonding. **a** A silane-modified polyacrylic acid (PAAc) hydrogel, with or without prestretch, is bonded to a silane-modified PDMS. Upon immersing the bilayer in a 0.1 M NaHCO_3_ solution, the PAAc hydrogel swells to ~9 times the original thickness. The swelling causes the hydrogel with prestretch to form a one-dimensional pattern, and causes the hydrogel without prestretch to form a two-dimensional pattern. The scale bar is 2 mm. **b** Co-drawing of an elastomer-coated hydrogel fiber. A fiber of oxygen-tolerant resin is extruded from a syringe (Supplementary Movie 4). The fiber is then dip-coated in a PDMS precursor. We keep both polymers uncrosslinked, and co-draw them into a thinner fiber. Subsequently, the coupling agents condensate to crosslink the networks and bond them. Subject to a uniaxial stretch *λ*, the hydrogel fiber length increases by a factor of *λ* while the diameter reduces by a factor of 1/*λ*^1/2^. **c** Digital images (up) and microscopic images (down) of an elastomer-coated hydrogel fiber. The fiber has an initial diameter ~1.4 mm, and is drawn to a fiber of diameter 154 µm. The scale bar is 500 μm. **d** SEM images showing the cross-sections of a PDMS-coated PAAm fiber. The scale bars are 20 μm (up) and 1 μm (down). **e** Dip-coat two layers of PAAm resins with PDMS coatings, stack them together, keep both polymers uncrosslinked, and press them into a thinner laminate. After curing, PDMS forms an insulating layer between two PAAm hydrogels, as well as a coating layer covering the surface. **f** Images of the cross-section of multilayered PAAm-PDMS structure. The scale bars are 2.5 mm (left) and 250 μm (right)

Our bonding approach also allows mechanical manipulations during the bonding and manufacturing processes. pH-responsive hydrogel can be prestretched before bonding to a PDMS substrate, resulting in aligned swelling patterns (Fig. 8a). The pH-responsive hydrogels are swollen to ~9 times their original thickness, with no debonding observed. With resins of suitable rheological properties, elastomers and hydrogels can be integrated like pastries of alternating layers of oil and flour. We demonstrate this concept with two primitive examples: co-drawing of a PDMS-coated PAAm fiber, and co-pressing of a multilayered PAAm–PDMS structure (Fig. 8b–f).

### Heat-resistant hydrogels

**Fig. 9:**
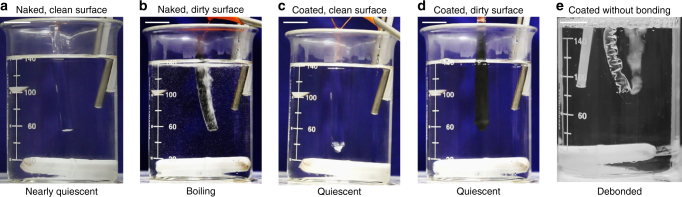
Deep-frying hydrogels in mineral oil at 120 °C. **a** A hydrogel of a clean surface remains nearly quiescent, except for a stream of bubbles emitting from a defect site. **b** When graphite powders are spread on the surface of a naked hydrogel, water vapor bubbles off from the entire surface. **c**, **d** Elastomer-coated hydrogels remain quiescent with or without graphite particles on the external surface. **e** If the elastomer is not silane modified, vapor bubbles easily nucleate underneath the coating, blow up the coating, and dehydrate the hydrogel. The scale bar is 2 cm

When a piece of food (i.e., a complex hydrogel) is deep-fried in oil, water vapor bubbles out. Such experience gives an impression that a hydrogel boils above 100 °C. Here we show that an elastomer-coated hydrogel readily survives elevated temperatures without boiling by immersing PAAm hydrogels with or without PDMS coatings in mineral oil heated at 120 °C (Fig. 9, Supplementary Movie 5).

A hydrogel boils either from inside or from surface. Boiling from inside needs to overcome capillarity, elasticity, and fracture energy^41–45^. A right combination of the cleanness, stiffness, and toughness of the hydrogel suppress the boiling from inside the hydrogel (Supplementary Note 1). In contrast, boiling from the surface only needs to overcome capillarity, but not elasticity or fracture energy. If the surface is clean, the water may be superheated without boiling (Fig. 9a). If the surface is contaminated with nucleation sites, boiling readily happens above 100 °C (Fig. 9b). Heat resistance of hydrogels is limited by the boiling from surface. In our experiment, we never observe bubbles form inside the hydrogel before bubbles form on the surface of the hydrogel.

We suppress the boiling from surface by a thin layer of bonded PDMS coating. The coating extends the barrier of elasticity and fracture energy beyond the surface of the hydrogel. The coated hydrogels do not boil with or without surface contamination (Fig. 9c, d). Suppressing the formation of bubbles requires strong adhesion between the hydrogel and the elastomer. If the elastomer is not silane modified and therefore does not bond with the hydrogel, vapor bubbles easily nucleate underneath the coating, blow up the coating, and dehydrate the hydrogel (Fig. 9e).

**Fig. 10:**
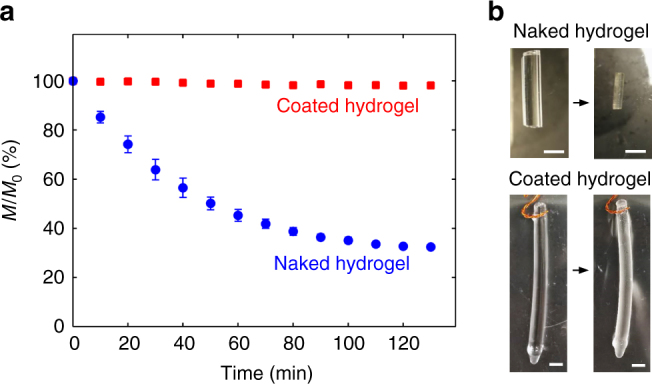
Weight loss of hydrogels deep-fried at 120 °C. **a** Normalized weight as a function of time for naked hydrogels and coated hydrogels deep-fried at 120 °C. The coated hydrogels maintain the weight while the naked hydrogels keep losing water over the frying time. After 130 min, the coated hydrogel preserves ~98% of its original weight, while naked hydrogel decreases to ~33% of its original weight; 33% weight corresponds to the dry polymer content in the hydrogel (*n* = 5). **b** Images of the hydrogels before and after frying. The scale bar is 1 cm

We further measure the weight of a sample as a function of the time of frying. We find that the coated hydrogel maintains ~98% of its original weight after 130 min of frying (Fig. 10), while the uncoated hydrogel is completed dehydrated.

Elastomer-coated hydrogels will enable applications at elevated temperatures, for example, as stretchable and transparent conductors to enable soft touchpads and displays for smart clothes that one can wear, wash, and iron. The capability will also enable hydrogels to integrate elastomers requiring high-temperature processes, such as vulcanization.

In summary, we report an approach to bond various hydrogels and hydrophobic elastomer for various materials in various manufacturing processes. We formulate oxygen-tolerant hydrogel resins for printing, coating, and drawing in the open air. We show that elastomer-coated hydrogels can sustain high temperature without boiling. It is hoped that this work will open doors to rapid-prototyping and mass-producing biomimetic hydrogel–elastomer devices for healthcare, fashion, and augmented reality.

## Methods

### Synthesis of silane-modified hydrogels

We dissolved AAm (Sigma-Aldrich A8887) in distilled water (Poland Spring) to form a solution of concentration 2 M. For every 1 ml of the solution, 4 μl of 0.1 M MBAA (Sigma-Aldrich M7279) is added as the conventional crosslinker and 20 μl of 0.1 M *α*-ketoglutaric acid (Sigma-Aldrich 75890) is added as the UV initiator. Unless otherwise specified, 1.9 μl of 3-(trimethoxysilyl) propyl methacrylate (TMSPMA, Sigma-Aldrich 440159) is added as the coupling agent. Since pre-hydrolysis TMSPMA is hydrophobic, the solution is vigorously stirred for 1 min to disperse, hydrolyze, and dissolve TMSPMA. The *α*-ketoglutaric acid makes the precursor acidic (pH ~3.5), which accelerates the hydrolysis of trialkoxysilane, but slows down the condensation of silanol groups; 33.3 μl of 0.3 M SDS (Sigma-Aldrich L3771) is added as surfactant. The solution is then poured into a mold made of laser-cut acrylic sheets (McMaster-Carr). The mold and the solution are covered with the bottom of a Petri dish to prevent oxygen inhibition. The covered mold is then placed under a UV lamp (15 W 365 nm; UVP XX-15L, 2 cm distance between sample and lamp) for half an hour to polymerize into the PAAm hydrogel. PAAc and poly(N-isopropylacrylamide) (PNIPAM) are prepared similarly using a 2 M solution of the corresponding monomer. The hydrogels are used as prepared so that the water content corresponds to the concentration of the precursor, which is 2 M AAm in water, unless otherwise specified.

### Synthesis of silane-modified PDMS

The precursor of the PDMS is made by mixing the base and the curing agent of Sylgard 184 (Dow Corning) at 10:1 weight ratio. Unless otherwise specified, 0.1% v/w of triethoxy(vinyl)silane (TEOVS, Sigma-Aldrich 175560) is then mixed into the precursor. The precursor is then left at ambient condition for 30 min for the air bubbles to float out, poured into a Petri dish, and cured at 65 °C in an oven (VWR, Model No. 1330GM) for 12 h. Silane-modified Ecoflex 0020 is prepared similarly using 0.3% v/w TEOVS.

### Synthesis of silane-modified polybutadiene

Ten percent w/w polybutadiene (Aldrich 181382) is dissolved in hexane (Sigma-Aldrich 34859); 0.1 M benzophenone (Sigma-Aldrich 139386) is dissolved in hexane. For every 10 g polybutadiene, add 9.26 μl 0.1 M benzophenone, 8.6 μl MPTMS, and 2.46 μl dibutyltin diacetate (Aldrich 290890). The solution is reacted under UV for 1 h. The solution is then poured into a Petri dish in a fume hood. After the hexane is evaporated, the rubber film is put in contact with a modified hydrogel. Bonding forms after 1 day.

### Measurement of adhesion

Samples of the PAAm hydrogel are prepared with the size of 90 × 20 × 3 mm. Samples of the PDMS elastomer are prepared with the size of 90 × 30 × 1.2 mm. The hydrogel is put on top of the elastomer right after curing unless otherwise specified. A 20 × 30 mm paraffin film (Bemis, Parafilm M) is inserted at the hydrogel–elastomer interface at one end. The film prevents the bonding in the corresponding area and serves as a pre-crack. The hydrogel–elastomer bilayer structure is stored at room temperature for 1 day for adhesion to develop. The sample is covered in a Petri dish to prevent the hydrogel from dehydration. The hydrogel–Ecoflex bilayer was stored at 65 °C for 1 day before test.

After 1 day in contact, the bilayer is taken out of the Petri dish. The elastomer side is bonded to a glass slide (VWR Catalog No. 48382-179) using a cyanoacrylate adhesive (krazy glue). For PDMS, the elastomer side is first bonded to a rubber band (McMaster-Carr, SBR) using silicone adhesive (Smooth-On, Sil-Poxy, 12 min for curing at room temperature) before bonding to the glass slide. The glass slide serves as the rigid substrate during the peeling test. The hydrogel side of the sample is glued to a thin polyester film (50.8 μm; McMaster Carr) using the cyanoacrylate glue. The polyester film serves as a flexible, inextensible backing for the hydrogel. If the hydrogel precursor is acidic, the surface of the hydrogel is first neutralized with a few drops of 0.1 M NaHCO_3_ (Sigma-Aldrich S5761) solution and dried with blowing air before applying the cyanoacrylate glue.

The sample with glass substrate and polyester backing layer is then loaded to a mechanical testing machine (10 N or 500 N load cells; Instron 3342 Single Column UTS) using the 90-degree peeling fixture (Catalog No. 2820-035). The peeling rate is 10 mm/min. The plateau value of the force–displacement curve gives the adhesion energy.

### Fabrication of a Fe-PAAc-toughened PAAm on PDMS

The silane-modified PDMS precursor is first spin-coated (Headway Research, PWM32-PS-R790) onto a glass slide (same type as used in other peeling tests) at 1000 rpm for 50 s. Since PDMS is rather permeable to oxygen, a thick layer of PDMS would act as a source of oxygen, and inhibit the curing of the second network in the hydrogel introduced later. The spin-coated thin layer of PDMS avoids this problem. The spin-coated sample is then cured as previously described for other PDMS samples. The hydrogel is fabricated and bonded to the PDMS as previously described as well.

After 1 day of bonding, the sample is immersed in the precursor of the second network in a Petri dish, which consists of 1.58 M of AAm, 0.2 M of AAc, and 0.004 M of *α*-ketoglutaric acid. After 1 day of immersion, the Petri dish with the immersed sample is covered with a polyethylene film (Minigrip, 2Mil) and polymerized under UV for 2 h. The polyethylene film prevents the oxygen inhibition of the polymerization. After the curing, the PAAm-co-AAc is not crosslinked. The excess PAAm-co-AAc outside the hydrogel is readily removed by a spatula. The sample is then immersed in a 0.06 M FeCl_3_ solution for 1 day. The Fe^3+^ ions diffuse into the hydrogel and crosslink the second network by forming coordination complex between the Fe^3+^ and the carboxyl groups of the AAc. The hydrogel turns brown after the crosslinking process. The sample is then taken out from the FeCl_3_ solution and immersed in distilled water for another day. Immersion in distilled water removes excess Fe^3+^ ions from the hydrogel and improves the quality of the crosslinking.

### Measurement of shear modulus

The shear moduli of PAAm and PDMS are measured by using a testing machine (10 N or 500 N; Instron 3342 Single Column UTS). PAAm samples are prepared with size 40 × 20 × 2 mm. The sample is stretched to 300% strain and the strain rate is 10 mm/min. PDMS samples are prepared with the size of 40 × 20 × 1.2 mm. The sample is stretched to 30% strain and the strain rate for uniaxial tensile test was 1 mm/min. The test data are then fitted with incompressible neo-Hookean model to obtain the shear modulus. If a hydrogel sample is tested multiple times, the sample is stored in a sealed Petri dish between tests to prevent dehydration.

### The effect of pH on the rate of condensation of silanols in PAAm hydrogels

The AAm hydrogel is prepared as described in the section “Synthesis of silane-modified hydrogels”. But the 2 M AAm solution is made with 0.1 M buffer solutions of different pH instead. The recipe for each buffer solution is listed in Supplementary Table 2.

### Fabrication of hydrogel–elastomer pneumatic actuator

We prepared a silane-modified PAAm, size 50 × 40 × 3 mm, and a silane-modified PDMS, radius 80 mm and thickness 1.2 mm. The PAAm is then laid onto the PDMS surface, sandwiching a circular film of paraffin, radius 20 mm and thickness 0.05 mm. The PAAm and the PDMS bond around the paraffin after 1 day. Then a nozzle is inserted into the unbonded region between the PAAm and the PDMS. Air is then pumped through the nozzle until the PAAm balloon blows up.

### Fabrication of a silane-modified PAAc hydrogel on top of PDMS

We fabricated a piece of PAAc following the same procedure as the modified PAAm, by replacing the 2 M AAm solution with a 2 M AAc (Sigma-Aldrich 147230) solution. A piece of modified PDMS is prepared as described in the section “Synthesis of silane-modified PDMS”. The modified PAAc is uniaxially stretched to three times its original length and clipped to an acrylic sheet. The modified PDMS is then laid over the prestretched hydrogel. The hydrogel–elastomer bilayer is kept in a sealed polyethylene bag (Minigrip) at room temperature for 1 day. The bilayer is then taken off from the acrylic sheet and submerged in 0.1 M solution of NaHCO_3_ until the hydrogel swells to equilibrium. For comparison, a hydrogel–elastomer hybrid sample without prestretch is prepared following a similar procedure.

### Fabrication of dip-coated G-clef

A silane modified PAAm hydrogel of the G-clef shape is prepared as before in a laser-cut mold. A Pt-catalyst for the curing of PDMS (Sigma Aldrich 479519) is added into the aforementioned silane-modified PDMS precursor at 0.1% v/w to accelerate the curing. The hydrogel G-clef is dipped in the PDMS precursor and hung at room temperature for half an hour to drain the excess PDMS. The G-clef is then dipped in the PDMS precursor for the second time followed by half an hour hanging. The sample is then lied in a Petri dish and sealed. The sample is left at room temperature for 1 day.

### Ionic circuit writing

Tetrahydrofuran (THF, Sigma-Aldrich 360589) is dehydrated with 10% w/v 4 Å molecular sieves (Sigma-Aldrich 208590) for 1 day. The chain transfer agent MPTMS (Sigma-Aldrich 175617) is diluted in the dehydrated THF to 1% v/v concentration. Conductive AAm precursor is made of 2 M AAm and 2 M NaCl solution. For every 1 ml 2 M AAm/NaCl solution, 10 μl of the 0.1 M acetic acid (Sigma-Aldrich A6283) is added to lower the pH; 2.85 μl of the 1% MPTMS in THF is then added. Afterward 1.9 μl TMSPMA is added. The solution is stirred for 1 min; 2.5 μl of 0.1 M Irgacure 2959 (Sigma-Aldrich 410896) is added as photo-initiator.

The solution is extracted into a plastic syringe (5 ml VWR 309646) with a blunt needle of 1.2 mm diameter. The syringe is placed under previously described UV setup for 30 min. The syringe seals the precursor from open air and prevents the oxygen inhibition during the polymerization. The syringe is then pressed to extrude the PAAm solution on a modified PDMS (Fig. 7c). The PDMS is modified with 2% v/v TEOVS and cured beforehand. Since the high ionic strength in the hydrogel interferes with the function of SDS, high amount of TEOVS is used to achieve bonding without surfactant. After the drawing, the sample is sealed in a Petri dish and left at room temperature for 1 day. For the drawing of the anglerfish, the sample is flushed with water soluble blue dye (VWR). The sample is then washed with distilled water to remove excess dye. This coloring process reveals the blue pattern. The upper half and the lower haft of the fish have been drawn as disconnected parts. A blue light emitting diode is inserted to connect the two disconnected parts at the head side. The tail side is connected to DC voltage source (Dr. Meter PS-305DM). The light is turned on with 6 V DC voltage.

### Printing of PAAm–PDMS mesh

The PAAm resin is prepared as described in the last section. PDMS resin is prepared by mixing every 1 g silane-modified PDMS (Sylgard 184 base: curing agent = 10: 1) with 1 μl Pt-catalyst (Sigma Aldrich 479519) to accelerate the curing. The mixture is extracted into a syringe and precured at 65 °C for 20 min before printing. The resin is printed into a mesh structure as shown in Supplementary Movie 3.

### Extrusion of PAAm hydrogel fiber

The preparation for the hydrogel resin is identical to the “Ionic circuit writing” section except that MPTMS is not added and a 0.9 mm diameter blunt needle is used instead of the 1.2 mm one. After 30 min UV curing, the syringe (5 ml VWR 309646) is compressed with the Instron machine (Instron3342 Single Column UTS) at fixed speed 5 mm/min. The fiber is collected by wrapping on a polypropylene vial (Falcon 50 ml polypropylene conical tube 30 × 115 mm style).

### Co-drawing of PDMS coated PAAm fiber

A PAAm fiber is spinned as described in the section “Extrusion of PAAm hydrogel fiber”. The fiber is directly coated with a silane-modified PDMS precursor. The fiber is then stretched and held in a sealed container at room temperature for 1 day. The fiber is then examined under optical microscope (Nikon, Me600) and SEM (Zeiss, FESEM ultra 55).

### Co-pressing of a multilayered PAAm-PDMS structure

Two layers of PAAm resin are prepared with the recipe of ionic circuit. A silane-modified PDMS precursor is prepared as before and is pre-cured at 65 °C for 20 min to increase the viscosity. One PAAm resin is placed on the bottom of a Petri dish wiped with a layer of PDMS precursor, followed with the wiping of another layer of PDMS on the top. Subsequently, the other PAAm resin is laminated. The multilayer is compressed by the cover of the Petri dish with a weight. The final thickness of the multilayer is controlled using two acrylic sheets as the spacers. After pressing, the multilayer is sealed inside a plastic bag, allowing the full curing of PDMS and condensation of silane for 1 day at room temperature.

### Stress relaxation test of the oxygen-tolerant hydrogel resin

The oxygen-tolerant hydrogel resins are prepared as before. The resins are polymerized in syringes of 18.8 mm inner diameter. The syringe is then cut open to take the sample out. The resins are cut into disk shape of roughly 18 mm thickness and put on the Instron machine. A pre-load of 0.02 N is applied to guarantee the contact between the sample and the load cell. The sample is then compressed to 50% its original height at a loading rate of 18 mm/s. The stress relaxation is then measured for 10 min.

### Deep-frying hydrogels

We used an 35% w/w AAm precursor to make the stiff hydrogel. For every 1 ml AAm solution, 200 µl 0.1 M MBAA is used. The resulting PAAm hydrogel has a shear modulus of 64.3 kPa, measured by the tensile test described before. To prepare a PDMS-coated hydrogel, the hydrogel is dipped into the precursor of PDMS five times. The resulting coating has a thickness of about 300 μm. Both the naked PAAm hydrogels and the PDMS-coated PAAm hydrogels are cured at 65 °C for 12 h in a sealed container.

A cup of mineral oil (VWR, BDH7338-4) is heated on a hot plate (VWR). A thermal couple is immersed in the oil and connected to the hot plate for the temperature control of the hot plate. A magnet stirrer is used to help homogenize the temperature in the oil. After the temperature of the oil stabilizes at 120 °C, the stirrer is stopped. The samples are hung on a steel bar via copper hooks, and fried in the oil for at least 5 min. The naked or coated hydrogel is dipped in graphite powder (Sigma-Aldrich, 282863) for dirty samples.

### Data availability

The data sets generated during the current study are available from the corresponding author on reasonable request.

## Electronic supplementary material

Supplementary Information

Peer Review File

Description of Additional Supplementary Files

Supplementary Movie 1

Supplementary Movie 2

Supplementary Movie 3

Supplementary Movie 4

Supplementary Movie 5
