# Supplementary material for: Bonding dissimilar polymer networks in various manufacturing processes
Source: Nat Commun. 2018 Feb 27;9:846. doi: 10.1038/s41467-018-03269-x (PMC5829243; doi:10.1038/s41467-018-03269-x)
Supplement: Supplementary file 1 — Supplementary Information [file 41467_2018_3269_MOESM1_ESM.pdf]

Supplementary Information

Supplementary Table 1 | Common organofunctional groups of silane coupling agents and polymers that can be modified

Coupling group R	Functional group to couple with	Possible polymers
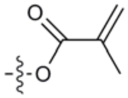	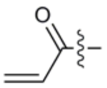 or 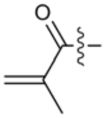	Acrylic resin,<br>Free-radical polymerized hydrogel
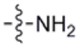	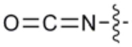	Polyurethane resin
	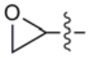	Epoxy resin (high temperature)
	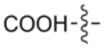	Alginate, Hyaluronate, Polyacrylic acid
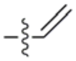	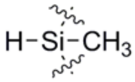	Addition-cured silicone
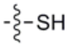	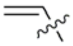 or 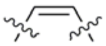	Unsaturated rubbers: natural rubber, butyl rubber, nitrile rubber, etc.
	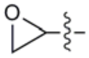	Epoxy resin (room temperature)

Supplementary Table 2 | Recipes for buffered hydrogel precursor

pH = 2	Acrylamide	Hydrochloric acid	Sodium chloride
	2M	0.01M	0.09M
pH = 4	Acrylamide	Acetic acid	Sodium hydroxide
	2M	0.1M	0.0148M
pH = 6	Acrylamide	Sodium phosphate dibasic	Sodium phosphate monobasic
	2M	0.0869 M	0.0131 M
pH = 8	Acrylamide	Sodium phosphate dibasic	Sodium phosphate monobasic
	2M	0.0062 M	0.0938 M

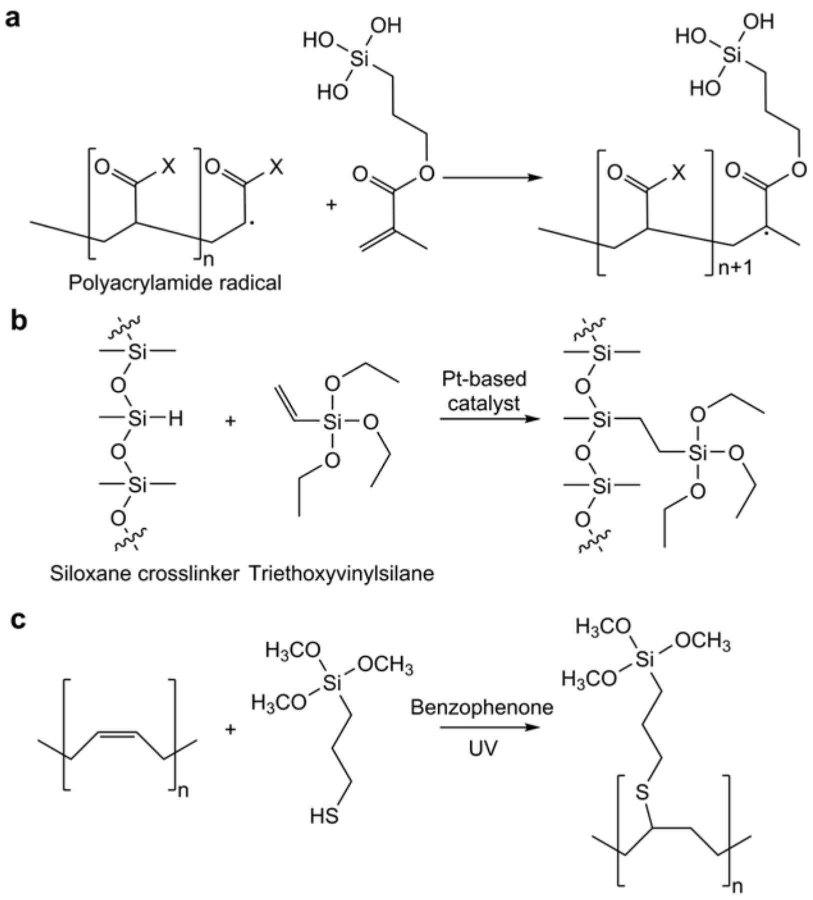

Supplementary Figure 1 | Incorporating silane coupling agent in different networks. **a**, In radical polymerized hydrogels. **b**, In Pt-catalyzed PDMS, i.e. Sylgard 184 and Ecoflex 0020. **c**, In polybutadiene. Silane coupling agent with other functional groups are commercially available, allowing the technique to be used in a much wider range of polymer systems than demonstrated in the current paper.

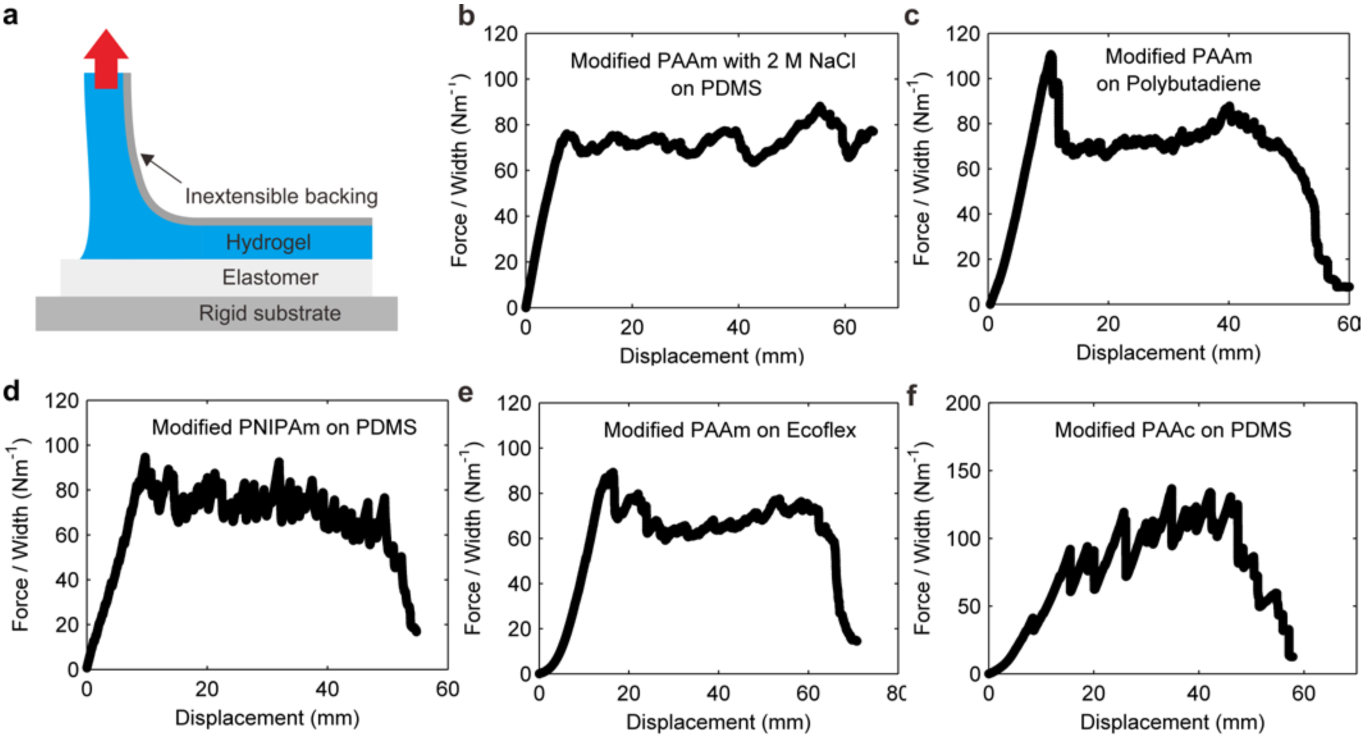

Supplementary Figure 2 | Peeling tests for different material pairs. a, Schematic of the setup of the peeling test. The representative force displacement curve for **b,** PAAm with 2 M NaCl on PDMS, **c,** PAAm on polybutadiene, **d,** PNIPAM on PDMS, **e,** PAAm on Ecoflex, and **f,** PAAc on PDMS. In these experiments, the concentrations of the coupling agents in both the hydrogel and the elastomer are about 0.1% vol. Assuming no segregation, the coupling agents are estimated to cover about 1% the area of the interface. The low density of bonds achieving strong adhesion is specific to stretchable networks. The forces in the networks are transmitted through crosslinks, and the bond density between the networks only need to be comparable to the crosslink density in the networks. So little coupling agents are required that our approach consumes much less trialkoxysilanes than a surface treatment[1] to achieve similar adhesion for samples of sizes on the order of 10 cm.

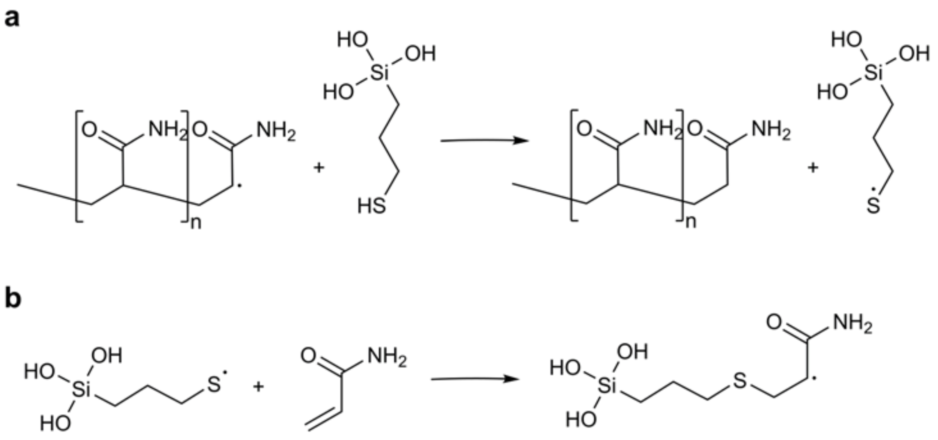

Supplementary Figure 3 | Chain transfer agent controls the viscosity of oxygen-tolerant resin by reducing the chain length. a, During the polymerization, chain transfer from a PAAm radical to the thiol group of a hydrolyzed MPTMS would end the propagating PAAm chain and generate a thiol radical. **b**, The thiol radical re-initiate a PAAm chain. In combination, each MPTMS cuts a propagating PAAm chain into two. As the average chain becomes shorter, the hydrogel resin becomes less viscous. The silanol groups on MPTMS at each chain end participate in the crosslinking through the condensation with other silanol groups.

Supplementary Note 1 | Analysis of heat resistance hydrogel

We interpret the observations of deep frying hydrogels as follows. The pressure of saturated water vapor is 101 kPa and 199 kPa at, respectively, 100°C and 120°C, setting up a differential pressure of 98 kPa between the vapor in a bubble-like defect inside the hydrogel and that in the ambient. The expansion of the bubble is resisted by capillarity, elasticity, and fracture energy. The surface energy of the hydrogel is comparable to that of water, $\gamma \sim 0.05 \mathrm{J/m^2}$ [2]. Taking the bubble to be comparable to the mesh size of the hydrogel, $a \sim 10^{-8}\,\mathrm{m}$ (discussed later), we estimate that the Laplace stress is $2\gamma / a$ ~ 10 MPa. The observed quiescence of much of the clean hydrogel indicates that large bubble-like defects are rare, so that water is superheated above its boiling point, in a state of metastability[3]. The effect is analogous to the rising of sap in a tree[4]. The graphite powder spread on the surface of the hydrogel reduces the nucleation barrier, so that the hydrogel boils readily.

To expand in the hydrogel, a bubble must also overcome elasticity, which requires an additional internal pressure of 2.5 times the shear modulus of the hydrogel[5]. Accordingly, the shear modulus of 64.3 kPa corresponds to a boiling temperature of 129 °C. This elastic constraint does not exist on the surface of the naked hydrogel, but increases to the full strength inside the hydrogel. The partial constraint of a near-surface nucleation site makes near-surface rupture of the naked hydrogel possible, as is happened in our naked hydrogel at about 90 seconds (Supplementary Video 5). In an elastomer-coated hydrogel, however, elasticity suppresses bubbling everywhere in the hydrogel and on the hydrogel-elastomer interface. The observed quiescence of the elastomer-coated hydrogels indicates that the elastomer coating is sufficiently stiff and adherent to suppress boiling on the elastomer-hydrogel interface. The quiescence of PDMS-coated hydrogel with graphite powers further indicates that water permeates through PDMS at a sufficiently low rate so that there is not enough water for surface boiling even with nucleation sites. An elastomer-coated hydrogel is analogous to a pressure cooker, elevating the boiling point of water by sustaining a higher internal pressure. Stiffer hydrogels will remain quiescent at higher temperatures.

The expansion of the vapor bubble can cause the hydrogel to fracture. The fracture of hydrogel is flaw size sensitive. If the bubble nucleates from a large defect, the hydrogel is fractured due to stress concentration before the pressure of 2.5 times shear modulus is reached. If the bubble nucleates from a small defect, the fracture toughness of the hydrogel would restrain the expansion of the cavitation above the pressure of 2.5 times shear modulus[6-8]. The critical length scale is identified as $0.1\Gamma/E$[6]. Here Γ is the fracture energy and E is Young's modulus. For our specific gel, the $0.1\Gamma/E$ threshold is 87 μm. Defect of 87 μm size is quite unlikely so the heat resistance at 129 °C is a conservative estimation. In principle, higher heat resistance can be realized using stiffer and tougher hydrogels.

The mesh size of the hydrogel is estimated as follows. According to Flory Huggins theory, the shear modulus of a swollen gel is related to the crosslinking density by $\mu = NkT\phi^{1/3}$ [9]. Here N is the crosslinking density in the dry state, k is Boltzmann's constant, T is the absolute temperature, and ϕ is the volume fraction of polymer in the gel. As we used 35% w/w AAm

precursor to synthesize the hydrogel for frying, $\phi \sim 0.35$. Room temperature, $kT \sim 4\times10^{-21}\,\mathrm{J}$. For $\mu = 64\,\mathrm{kPa}$ we have $N \sim 2\times10^{25}\,\mathrm{m}^{-3}$ and mesh size ~ $\left(\phi N\right)^{1/3} \sim 2\times10^{-8}\,\mathrm{m}$.

Supplementary References

1. Yuk, H., Zhang, T., Lin, S., Parada, G. A., Zhao, X. Tough bonding of hydrogels to diverse non-porous surfaces. *Nat. Mater.* **15**, 190-196 (2016).
2. Lemmon, E. W., McLinden, M. O., Friend, D. G. Thermophysical Properties of Fluid Systems. In: *NIST Chemistry WebBook*. National Institute of Standards and Technology.
3. Caupin, F., Herbert, E. Cavitation in water: a review. *C. R. Phys.* **7**, 1000-1017 (2006).
4. Holbrook, N. M., Zwieniecki, M. A. Transporting water to the tops of trees. *Phys. Today* **61**, 76-77 (2008).
5. Gent, A. N., Tompkins, D. A. Nucleation and growth of gas bubbles in elastomers. *J. App. Phys.* **40**, 2520-2525 (1969).
6. Gent, A. N., Wang, C. Fracture mechanics and cavitation in rubber-like solids. *J. Mater. Sci.* **26**, 3392-3395 (1991).
7. Williams, M. L., Schapery, R. A. Spherical flaw instability in hydrostatic tension. *Int. J. Fracture Mech.* **1**, 64-72 (1965).
8. Cristiano, A., *et al.* An experimental investigation of fracture by cavitation of model elastomeric networks. *J. Polym. Sci. Part B: Polym. Phys.* **48**, 1409-1422 (2010).
9. Hu, Y., Chen, X., Whitesides, G. M., Vlassak, J. J., Suo, Z. Indentation of polydimethylsiloxane submerged in organic solvents. *J. Mater. Res.* **26**, 785-795 (2011).
