# Supplementary material for: Bonding dissimilar polymer networks in various manufacturing processes
Source: Nat Commun. 2018 Feb 27;9:846. doi: 10.1038/s41467-018-03269-x (PMC5829243; doi:10.1038/s41467-018-03269-x)
Supplement: Supplementary file 3 — Description of Additional Supplementary Files [file 41467_2018_3269_MOESM3_ESM.pdf]

**Description of Additional Supplementary Files**

**File Name: Supplementary Movie 1**
**Description:** Inflating a hydrogel balloon adherent on an elastomer. A piece of silane-modified PAAm hydrogel and a piece of silane-modified PDMS are molded separately. The PAAm is then laid on top of the PDMS with a circular paraffin film in between. After one day at room temperature, the PAAm is bonded with the PDMS outside the area covered by the paraffin film. Then a nozzle is inserted in between the hydrogel and the PDMS to blow the hydrogel to several times of its original size. The balloon eventually ruptures by the fracture of the hydrogel layer rather than debonding of the interface.

**File Name: Supplementary Movie 2**
**Description:** Dip-coating a thin layer of elastomer outside a hydrogel of arbitrary shape. A silane-modified PAAm hydrogel is molded in the shape of G-clef, and is dip-coated with a thin layer of silane-modified PDMS precursor. Then the sample is hung in a sealed container at room temperature for one day for the silanols to condensate. Then the hydrogels are immersed in colored distilled water for 20 seconds, and then rinsed in colorless DI-water. The dip-coated hydrogel is still transparent and colorless, while the uncoated hydrogel is colored. Then the dip-coated hydrogel is stretched and rubbed for seconds, and it recovers to its original shape without debonding.

**File Name: Supplementary Movie 3**
**Description:** Printing hydrogels and elastomers. Silane-modified PAAm hydrogel resin is colored with blue dye and extruded on a Petri dish. Partially cured PDMS resin is then extruded into lines perpendicular to the hydrogel lines. The mesh is covered in a Petri dish and cured at 65°C for 12 hours. The mesh can then withstand stretching and twisting.

**File Name: Supplementary Movie 4**
**Description:** Spinning a hydrogel fiber. The silane-modified PAAm hydrogel resin with a higher viscosity is first prepared in a 5 mL syringe. Then the syringe with a blunt needle of 1.2mm diameter is compressed with the mechanical test machine at fixed speed 10 mm/min. The fiber is extruded in a uniform speed and collected by wrapping on a polypropylene vial.

**File Name: Supplementary Movie 5**
**Description**: Deep-frying hydrogels. Naked PAAm hydrogels and PDMS-coated PAAm hydrogels are deep-fried in mineral oil at 120 0C. The mineral oil is heated on a hot plate. The temperature of mineral oil is monitored using a temperature sensor. The samples are hung on a steel bar via copper hooks. After the temperature of mineral oil stabilizes at 120 0C, samples are immersed into the mineral oil for at least 5 minutes. During deep-frying, a coated hydrogel can be twisted. For a hydrogel coated with a PDMS containing no silane, water vapor bubbles readily nucleate underneath the coating, blow up the coating, and dehydrate the hydrogel.
