# Supplementary material for: Bonding dissimilar polymer networks in various manufacturing processes
Source: Nat Commun. 2018 Feb 27;9:846. doi: 10.1038/s41467-018-03269-x (PMC5829243; doi:10.1038/s41467-018-03269-x)
Supplement: Supplementary file 2 — Peer Review File [file 41467_2018_3269_MOESM2_ESM.pdf]

Editorial Note: This manuscript has been previously reviewed at another journal that is not operating a transparent peer review scheme. The manuscript was considered suitable for publication without further review at *Nature Communications.*
